# Dr. Hull's Answer to Dr. Ferriar

**Published:** 1801-02

**Authors:** John Hull

**Affiliations:** Manchester


### *To the Editors of the Medical and Phyſical Journal.*

GENTLEMEN,

THE laſt Number of your very uſeful Journal contains an intemperate letter, addreſſed by Dr. Ferriar, of this town, to Dr. Batty, in which the Doctor attempts to vindicate himſelf againſt two charges, publiſhed in my Eſſay on Phlegmatia Dolens, lens, namely, of *borrowing* his account of the theory and cure of this difeafe from Mr. Trye's *Essay on the swelling of the lower extremities incident to lying-in women*, and of *misrepresenting* the cafe of Jane Waters.

The remarks I fhall now make upon the Doctor's letter, will, I doubt not, convince your numerous readers, that both the charges are well founded, and that the Doctor would have acted more prudently, if he had not publifhed his vindication.

Dr. Ferriar declares, that he derived his opinion of the nature of the difeafe from the evidence of his fenfes, without obligation to any preceding writer, and that his method of cure was directly deduced from that opinion: He further declares, that he has never *read* Mr. Trye's publication, which I have mentioned as the fource of his opinion; but he acknowledges that he had heard fome account of it; for he fays, "I underftood, however, at the time of publifhing my Obfervations, that he" (Mr. Trye) "confined his views entirely to the difeafe in the puerperal ftate."

That the Doctor has actually *read* Mr. Trye's Effay I cannot prove by pofitive teftimony; but I am able to adduce a chain of circumftantial evidence, which, I think, will leave no doubt on the minds of your readers, that he has at leaft *peeped* into the book, and has gleaned from it the principal doctrines contained in his own paper. Mr. Trye's work is in the Library of the Manchefter Infirmary; and I have good authority for faying, it is the uniform practice to circulate the pamphlets on medical fubjects amongft the Faculty of that Charity, foon after their introduction into the library. Hence it would appear, that this book has been *once* thruft into Dr. F's hands, and it has been *very often* before his eyes: And when we confider with what eagernefs the Doctor has hunted out curious and ludicrous extracts, in order to cram them into his indecent *Illustrations of Sterne*, it feems highly improbable, that, when he was engaged in writing his difingenuous attack on Mr. White's Theory of Phlegmatia Dolens, he would omit confulting the only other book at that time written in the Englifh language exprefsly on the fubject of this difeafe. Moreover, the points of coincidence in Mr. Trye's and Dr. Ferriar's Effays are fo numerous, that, in my opinion at leaft, this circumftance alone is fufficient to prove them to be *not accidental.* Mr. Trye has endeavoured to prove, 1ft. *That Mr. White's theory of the disease is not well founded:* 2dly. *That the proximate cause of the disease is an inflammation of the lymphatic glands or vessels:* 3dly. *That the disease occurs in persons, independently of parturition:* And, 4thly. He has pointed out a *method of cure, founded on his own opinion of the nature of the disease, which consists in the application of leeches and blisters to*

the

*the limb, &c.* Dr. Ferriar has gone over the fame ground, after the fhort interval of about fix years, and yet contends for the originality of his own doctrines.! When I gave an Analyfis of Dr. F's Effay, it did not occur to me that the Doctor confidered as an *original suggestion*, his opinion that the difeafe fometimes happens to perfons not in the puerperal ftate; otherwife I fhould *then* have proved, that Mr. Trye had anticipated him in this as well as in every other material circumftance. — Permit me, therefore, to lay before your readers the annexed paffage, from the 45th page of Mr. Trye's Effay: "Laftly, this kind of fwelling of the leg, thigh, and buttocks, is not peculiar to women who have fuffered parturition; I have feen it come on, nearly in the manner which Mr. White defcribes, in a perfon who had fuffered greatly from a retention of urine and inflammation of the bladder. No circumfcribed tumour could be felt in the iliac regions, therefore it is probable that there was a fwelling of the internal iliac glands, excited by fome acrid matter abforbed from the bladder. The difeafe was cured by frictions of mercurial ointment."

I am much deceived, if I have not fatisfactorily fhown in another place,* that Dr. Ferriar did not find, as he has afferted, the lymphatic veffels inflamed or enlarged in the limb of Jane Waters. Whether the fuperficial lymphatics were inflamed, enlarged, and entwifted like bundles of cord, in the limb of the anonymous gentleman whofe cafe he has related, may with reafon be doubted. It is generally underftood by the profeffional gentlemen at Manchefter, that Mr. T. W. of P. is the gentleman alluded to by Dr. Ferriar; and if this opinion be correct, I am informed that the limb has ulcerated fince the Doctor attended him, and that the Doctor has miftaken the limb in his Report. How many other errors he may have committed, with refpect to this cafe, I cannot determine; perhaps he may have miftaken varicofe veins for lymphatics!

Having now, I prefume, fubftantiated the charge of PLAGIARISM, by *circumstantial evidence*, as completely againft Dr. Ferriar as he himfelf has done againft the celebrated author of Triftram Shandy, I fhall next proceed to prove, by *positive evidence*, that he has moft grofsly MISREPORTED the Cafe of Jane Waters.

Dr. F. ftates in his letter, that his Report on the 21ft of January runs thus: "The affection of the limb is completely removed. She has no complaint excepting debility, and a cough which fhe had contracted previously to her lying-in." To eftablifh the accuracy of this Report, he tells us, that "Home-

* See Effay an Phlegmatia Dolens, p. 107.

" Home-patients of the Manchefter Infirmary are thofe who are attended at their own houfes;" that " Out-patients are thofe who are able to come for their medicines to the Difpenfary on ftated days." That " on referring to the lift of home-patients, he finds Jane Waters was made an out-patient on the 27th of January, 1798;" and that " the evidence of the home-patient book muft prove, that fhe was able to walk abroad on the 27th of January, 1798." This is the point at iffue betwixt us. I contend that the Doctor's definition of an out-patient is incomplete; that there are out-patients of the Manchefter Infirmary, who are not able to go for their medicines, and that the evidence of the home-patient book does not by any means warrant the Doctor's conclufion. I have already fhewn, (Effay, p. 112,) from the pofitive declaration of Jane Waters, that whilft fhe remained in Manchefter, which was about a month after her delivery, the fwelling of her limb did not abate materially, nor did fhe attempt to clean out her room," and that " fhe was never able to walk, or ftand, whilft fhe ftaid in Manchefter." To her own teftimony, I now beg leave to add that of her hufband, as tranfmitted to me in the following note.

" Mr. Tomlinfon prefents his refpects to Dr. Hull, and informs him that, agreeably to his requeft, he has this day called upon Jane Waters, of Afhley Yard, Fennel Street, Manchefter; that fhe has given him the fame account of the circumftances of her cafe, that is ftated by Dr. Hull; and her hufband has alfo declared to him, *that she never was able to walk during her stay in Manchester, after the swelling of her limb came on*; but that fhe was carried down ftairs on Monday, the 22d of January, 1798, in order to be placed in the coach, which conveyed her to her father's houfe at Newton Heath, and that fhe remained unable to walk for eleven weeks longer, when the abfcefs in her loins broke, and fhe gradually recovered."

*King Street, Jan.* 8, 1801. (A Copy.)

It may be proper to obferve here, that Newton Heath is at the diftance of three miles from Manchefter; if, therefore, fhe continued to be a patient of the Manchefter Infirmary, fhe muft neceffarily be an *out-patient*, as fhe was removed far beyond the limits within which the phyficians are required to vifit home-patients.

Dr. Ferriar fays, that in oppofition to the *facts* ftated by him, I have produced a " narrative without fome effential dates," and he is " *sorry to say*, without probability." The Doctor's

Doctor's objection to a want of dates is, I truſt, ſatisfactorily obviated by the above note: And I think he has committed an error in obſerving, that he is *sorry to say my narrative is without probability.*

To invalidate the evidence of Jane Waters, he calls her a poor ignorant creature, and intimates, that her recollection of the circumſtances of her complaint muſt neceſſarily be *confused* at the diſtance of nearly two years. To obviate the latter objection it may be ſufficient to obſerve, that I have learnt within theſe few days, from a profeſſional gentleman of great eminence, and on whoſe veracity I have a perfect reliance, that ſhe gave *him* an account of her caſe, very ſoon after her recovery, which agrees with that publiſhed in my Eſſay on Phlegmatia Dolens, and is, conſequently, contradictory of Dr. Ferriar's report in the moſt eſſential circumſtances. That Jane Waters is ignorant, compared with the Doctor, muſt be allowed; but ſhe appears to be as intelligent as women in her ſituation uſually are, who in general remember extremely well the circumſtances attending their labours and lyings-in, theſe being amongſt the moſt important occurrences of their lives. And here let me aſk, whether learning is requiſite to enable a woman to ſpeak accurately to ſuch circumſtances as are given in my ſtatement of her caſe? Will a Court of Law require, that a woman ſhould have read Valentini Novellæ Medico-Legales, Taliacotius de Curtorum Chirurgia, Beroalde, Bruſcumbille, &c. &c. before ſhe can be enabled to tell how many months elapſed after delivery, before the ſwelling of her limbs ſubſided, and ſhe regained the power of walking? Will the Court of Honour, to which the Doctor has poured out his pitiable complaints, deem it neceſſary, that Jane Waters ſhould be as deeply read as Dr. Ferriar, in order to tell truly, whether the ſwelling of her limb was removed in three weeks, or in four months? I preſume they will not. Will Dr. Ferriar, though aided by his notes, and the evidence of the Home-patient book, (which he muſt know does not prove what he has contended for) aſſert, in oppoſition to the poſitive evidence of two credible witneſſes, that Jane Waters was able to walk to the Infirmary on the 27th of January, 1798? Will he contend, that ſhe was able to walk abroad on that day? Will he perſiſt in ſaying that ſhe was ever able to walk acroſs her room, whilſt ſhe was under his care?

I ſhall conclude my remarks upon this part of the Doctor's letter, by ſtating, that I believe Jane Waters's account of her own caſe, as publiſhed in my Eſſay, will by the generality of readers be deemed correct, whilſt the Doctor's report will be regarded as ſmelling ſtrongly of the *Farrier's* ſhop, where the foot is not uncommonly adapted to the ſhoe, inſtead of the ſhoe being adapted to the foot.

One part of Dr. Ferriar's letter ſtill remains to be attended to. He *denounces* my Inquiry into the caſe of Jane Waters as an offence of a moſt ſerious nature, and declares, that I ſhew a total diſregard to profeſſional character by an avowal of it. When the conſequences of this inquiry are conſidered, the Doctor's denunciation is not to be wondered at. He muſt neceſſarily be galled to find it proved to the medical world, that his hiſtory of the caſe is groſſly miſreported, and that his *original* opinions, relative to the cure, theory, &c. of Phlegmatia Dolens, were publiſhed ſix years before, by Mr. Trye.

Towards a man, who had enliſted under the banners of Mr. Simmons, and attacked me in the manner he has done, I do not think it neceſſary to conduct myſelf with more delicacy, or leſs ſeverity. If Dr. Ferriar ſhould henceforward be deemed a Copyiſt, and his Medical Hiſtories be conſidered as wanting the ſtamp of authenticity, he has only himſelf to blame. He ſhould have repreſſed the ill-natured and ill-founded Remarks, which he made upon the Second Part of my Obſervations on Mr. Simmons's Detection, when voted into the library of the Mancheſter Infirmary, if he expected, or wiſhed, the veil not to be withdrawn from his errors.

An, ſi quis atro dente me petiverit,

Inultus ut flebo puer? HOR. Epod.

*Mancheſter, Jan.* 13, 1801. JOHN HULL.

